# The influence of onset of disease on exit from paid employment among workers in The Netherlands

**DOI:** 10.1093/eurpub/ckac129.438

**Published:** 2022-10-25

**Authors:** RW Hijdra, SJW Robroek, A Burdorf, M Schuring

**Affiliations:** Public Health, Erasmus Medical Center, Rotterdam, Netherlands

## Abstract

**Background:**

This study investigates the influence of onset of disease on exit from paid employment, and whether this differs across diseases and sociodemographic groups.

**Methods:**

Register data from Statistics Netherlands on medication prescription was linked to information on employment status and demographics. Persons who were employed in 2009 and 2010, and who did not use medication for the selected disease in 2009 (n = 5,889,036) were followed-up during 9-years. Six diseases were identified based on medication prescription in 2010 and 2011: cardiovascular diseases, inflammatory diseases, diabetes mellitus, respiratory diseases, psychological disorders, and psychotic disorders. Four pathways out of paid employment were defined: disability benefits, unemployment, no income, and early retirement. Cause-specific Cox Proportional Hazards regression analyses were performed, with interaction terms for age, sex, and migration background.

**Results:**

Onset of disease increased the likelihood to exit paid employment, with strongest associations for psychotic disorders (HR 2.91, 95% CI 2.81-3.02) and psychological disorders (HR 2.01, 95%CI 1.98-2.04). Onset of disease was most strongly associated with disability benefits, followed by unemployment. The influence of psychological and psychotic disorders on disability increased until around middle-age, after which it decreased. The influence of mental health problems on exit from paid employment was stronger for persons with a non-native Dutch background and males.

**Conclusions:**

Onset of diseases, especially mental health disorders, is a risk for early exit from paid employment. Effective interventions are needed to enhance an inclusive workforce and prevent involuntary loss of paid employment.

**Key messages:**

• Onset of all diseases increased the likelihood of exiting paid employment, through disability benefits, followed by unemployment.

• Onset of psychological and psychotic disorder had the strongest association with exiting paid employment, especially among males and workers with a migration background.

